# Spatial genetic diversity in the Cape mole-rat, *Georychus capensis*: Extreme isolation of populations in a subterranean environment

**DOI:** 10.1371/journal.pone.0194165

**Published:** 2018-03-15

**Authors:** Jacobus H. Visser, Nigel C. Bennett, Bettine Jansen van Vuuren

**Affiliations:** 1 Centre for Ecological Genomics and Wildlife Conservation, Department of Zoology, University of Johannesburg, Auckland Park, South Africa; 2 Mammal Research Institute, Department of Zoology and Entomology, University of Pretoria, Pretoria, South Africa; National Cheng Kung University, TAIWAN

## Abstract

The subterranean niche harbours animals with extreme adaptations. These adaptations decrease the vagility of taxa and, along with other behavioural adaptations, often result in isolated populations characterized by small effective population sizes, high inbreeding, population bottlenecks, genetic drift and consequently, high spatial genetic structure. Although information is available for some species, estimates of genetic diversity and whether this variation is spatially structured, is lacking for the Cape mole-rat (*Georychus capensis*). By adopting a range-wide sampling regime and employing two variable mitochondrial markers (cytochrome *b* and control region), we report on the effects that life-history, population demography and geographic barriers had in shaping genetic variation and population genetic patterns in *G*. *capensis*. We also compare our results to information available for the sister taxon of the study species, *Bathyergus suillus*. Our results show that *Georychus capensis* exhibits low genetic diversity relative to the concomitantly distributed *B*. *suillus*, most likely due to differences in habitat specificity, habitat fragmentation and historical population declines. In addition, the isolated nature of *G*. *capensis* populations and low levels of population connectivity has led to small effective population sizes and genetic differentiation, possibly aided by genetic drift. Not surprisingly therefore, *G*. *capensis* exhibits pronounced spatial structure across its range in South Africa. Along with geographic distance and demography, other factors shaping the genetic structure of *G*. *capensis* include the historical and contemporary impacts of mountains, rivers, sea-level fluctuations and elevation. Given the isolation and differentiation among *G*. *capensis* populations, the monotypic genus *Georychus* may represent a species complex.

## Introduction

The life history of a species along with its habitat specificity, habitat matrix and the spatial and temporal variation in geological and climatic factors through evolutionary time, shape genetic patterns and consequently drives diversification and speciation (e.g., [1, 2, 3, 4]). As such, dispersal capability and population dynamics impinge on genetic diversity and spatial genetic structure.

The subterranean niche is a relatively stable, predictable, and highly specialized domain with preferred habitat patches often in a disjunct distribution [5]. Not surprisingly, fossorial/subterranean taxa show extreme adaptations to burrowing and respiration, linked to fundamental changes in molecular and physiological pathways, behaviour and morphology [5]. As a result, fossorial species typically have low vagility and exhibit behavioural attributes such as territoriality and life-long fixed home ranges ([6, 7, 8, 9, 10, 11, 12], but see [13, 14]). Populations of fossorial species therefore regularly exhibit a localized and patchy distribution adhering to specific soil types [5, 7, 9, 10, 15, 16, 17, 18, 19] with restricted gene-flow between them [4, 9, 17]. Given such spatial isolation and often small effective population sizes, fossorial animals are prone to inbreeding and typically experience bottlenecks and genetic drift, which in turn leads to low genetic diversity and rapid divergences between isolated populations [4, 5, 7, 9, 10, 11, 12, 17, 20, 21, 22, 23, 24].

One species for which genetic information is still lacking is the Cape mole-rat, *Georychus capensis* [25]. The Cape mole-rat is a solitary, territorial subterranean species [26, 27, 28] which extends their burrow systems in search of food and mates [29, 30] and use seismic signalling (hindfoot drumming) during mate attraction [27, 28, 31]. *Georychus* is characterized by a disjunct distribution across its South African range (Western Cape, south-western KwaZulu-Natal and Mpumalanga Provinces, [19, 31, 32, 33, 34, 35] and is restricted to areas where specific ecological variables are present. This species remains largely understudied. Most recently, a study by Visser *et al*. [19] documented large geographic differences between populations with regards to mating variables. *Georychus* is considered monotypic [36, 37] with currently available molecular and allozyme data suggesting that populations from KwaZulu-Natal [38, 39, 40] and Mpumalanga [41] may be a separate species from those in the Western Cape Province.

In this study, we investigate the effects of life-history characteristics on shaping genetic variation and spatial genetic patterns in *G*. *capensis*. Our specific aims are to 1) document genetic diversity at the population level as well as across the range of *G*. *capensis*, 2) investigate demographic stability (both within populations and across the range), 3) test whether genetic isolation is present across the disjunct distribution, 4) compare our results to a previous study on *Bathyergus suillus* (the sister taxon of *G*. *capensis*; separated for 20–26 Mya, [20]) with regards to the genetic diversity and genetic structure across the same broad distribution in the Western Cape and lastly 5) identify possible geographic barriers to gene-flow across the distribution of *G*. *capensis*. We employed two variable mitochondrial markers, cytochrome *b* and the control region fragment. Mitochondrial markers are suitable for studying phylogeographic patterns and provide valuable baseline data regarding long-term isolation.

Investigations of genetic patterns across the range of a species allows for the identification of specific regions of possible conservation focus, as well as insight into the evolution of the Bathyergidae in general. The Cape mole-rat is presently a least concern species [42], but this assessment was based on limited information, and any novel information regarding genetic structure and hence possible taxonomic revisions, and demographic changes may inform its conservation status. Genetic diversity should be considered in conservation planning [43] as it is necessary for the long-term sustainability of populations through allowing adaptation to changing environments (population fitness, [44, 45, 46, 47]). Decreases in population size may lead to inbreeding and/or genetic drift, reduced fitness and adaptability [45].

## Materials and methods

### Sample collection

**Fig 1:**
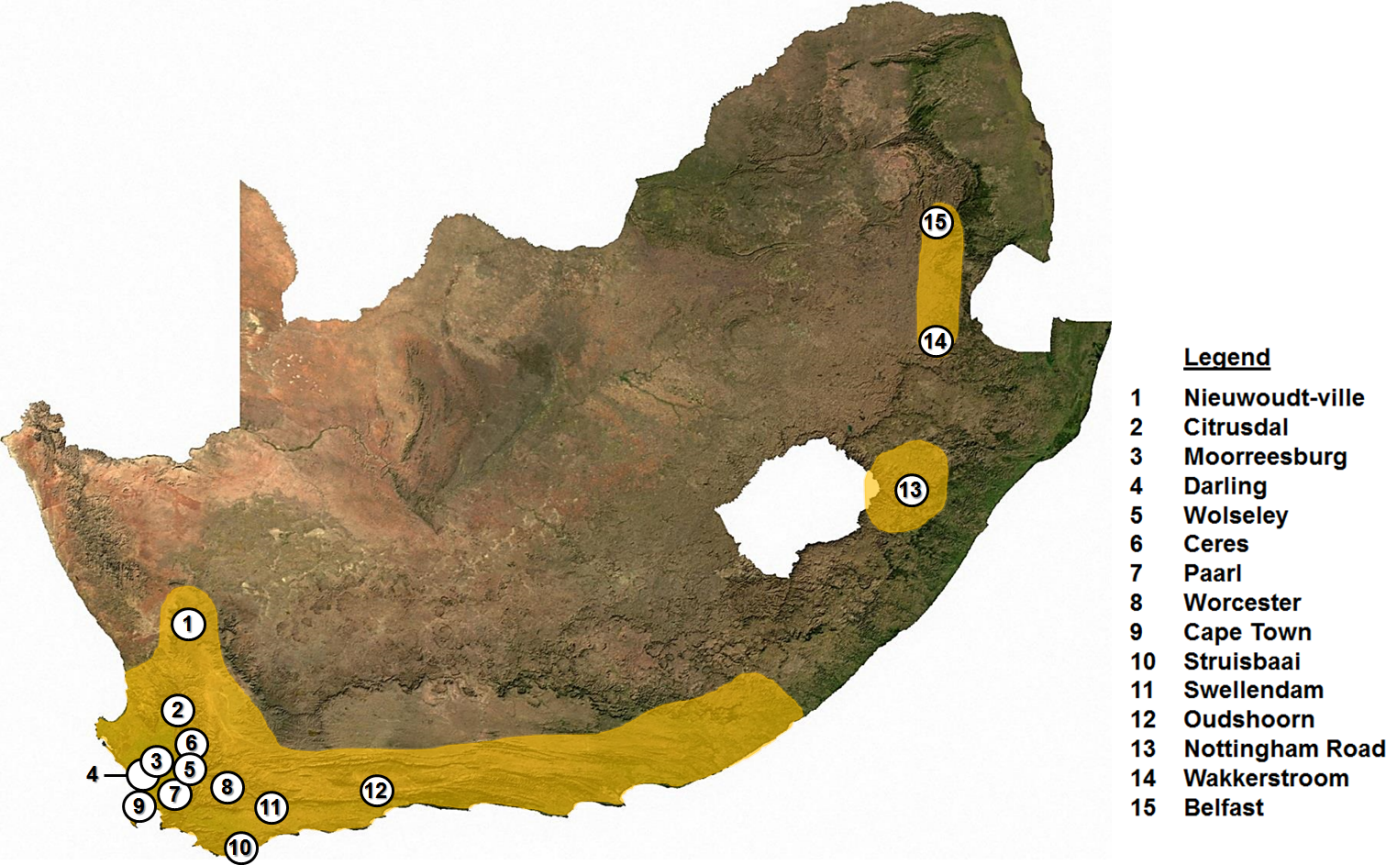
*Georychus capensis* sampling localities. Map showing localities where *G*. *capensis* were sampled across South Africa. The South African range (yellow shaded) of the species is indicated (based on historical records).

*Georychus capensis* specimens were collected from 15 localities (Fig 1) across its known distributional range in the Western Cape (Cape Nature Permit Number: 0056-AAA041-00084), KwaZulu-Natal (EKZNW Permit Number: OP1716/2016) and Mpumalanga Provinces (MPTA Permit Number: 5524), South Africa. In total, 265 *G*. *capensis* were collected by placing Gophinator traps (US patent No. 7,380,368, commercially available from Trapline Products, Menlo Park, California, USA) baited with peanut butter inside the burrow systems. The trap works in a similar way to the Victor Easy Set or Macabee traps, however uses a more powerful torsion mechanism which draws the animal into a pivot point at the front of the trap and applies ample power and pressure to quickly, consistently and humanely sacrifice rodents in the body mass- and size class of *G*. *capensis* (200 mm long and 400 g in mass). Collection of specimens was performed under ethical clearance from the Ethics Committee of the University of Johannesburg (Ethics number 215086650-10/09/15). Traps were checked every hour and the sacrificed animals were removed and immediately frozen at -10°C.

### DNA extraction and sequencing

Total genomic DNA was extracted from tissue (tail clippings) using a commercial DNA extraction kit (DNeasy Tissue and Blood kit; Qiagen) following the manufacturer's protocol. Two mitochondrial DNA fragments were targeted during PCR amplifications and sequencing using universal primers: 976 bp of the protein coding cytochrome *b* gene (*L14724* and *H15915*; [48, 49]) and 843 bp of the hypervariable control region (*LO* and *E3*; [50]).

PCR amplifications followed standard protocols (see [51]). Amplifications were performed in a MultiGene Optimax system (Labnet International, Inc.) at fragment-specific annealing temperatures (50°C for cytochrome *b* and 48°C for the control region). Amplicons were sequenced using BigDye chemistry following the protocols outlined in Jansen van Vuuren and Chown [52]. Electropherograms of the raw data were aligned and checked manually (Geneious Pro™ 7.1.7 software; Biomatters Ltd, New Zealand).

### Data analyses

#### Summary statistics and, population demography

The two mitochondrial fragments were analysed separately (cytochrome *b* accession numbers: MG496663—MG496927; control region accession numbers: MG496398—MG496662) as well as in concert (1819 bp); the results were largely congruent between fragments and the concatenated segments. Summary statistics (number of haplotypes, haplotype diversity and nucleotide diversity) for each population was calculated in Arlequin version 3.5 [53]. Possible fluctuations in population size were investigated using Fu’s Fs in DnaSP 5.10.01 [54]. To determine a measure of effective population sizes, Θ (theta) values for populations were calculated in Migrate 3.6.4 [55] using a Bayesian search strategy. Result were based on averaging over three replicates of 15 000 000 generations each (burnin = 3 000 000).

#### Population analyses

**Fig 2:**
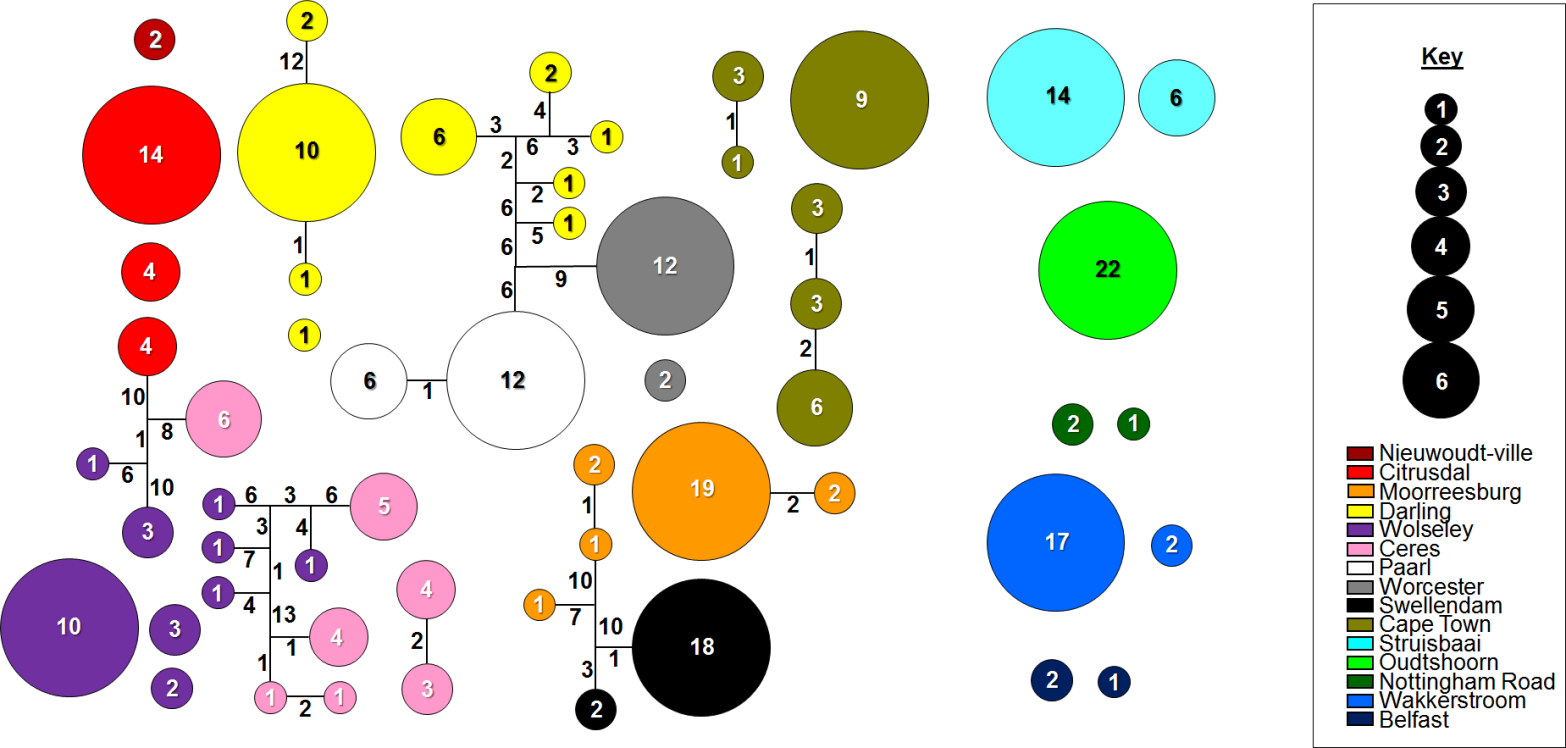
*Georychus capensis* haplotype network (combined dataset). Haplotype network, constructed from the combined dataset, for all *G*. *capensis* populations. The size of each circle reflects the number of specimens (also indicated in the circles) that share a particular haplotype. Numbers on branches represent the number of mutational steps separating haplotypes. Separate haploclades reflect haplotypes which differ by more than 18 mutational steps form other haplotypes.

As the majority of sampling localities were in the Western Cape, two separate datasets were constructed to account for sampling bias: 1) all sampled localities (localities 1–15 as shown in Fig 1) and 2) only localities in the Western Cape (localities 1–12 on Fig 1, hereafter referred to as the Western Cape region). To ascertain whether genetic diversity is significantly partitioned across the sampled range, overall Ф_ST_ values were calculated using an AMOVA considering all the sampling localities (l—15) together. To assess the impact of range fragmentation on the spatial genetic diversity, we divided the samples into three groups (localities 1–12, locality 13, and localities 14–15; these groupings correspond to the three provinces namely Western Cape, KwaZulu-Natal, and Mpumalanga). Finally, we assessed whether genetic diversity was significantly structured across the Western Cape region (localities 1–12). Additionally, pairwise Ф_ST_ values were calculated between all sampled localities. Significance for these tests were determined through 9 999 permutations of the data (Arlequin version 3.5; [53]). Isolation-by-distance was evaluated for the Western Cape region using a Mantel test as implemented in Alleles In Space version 1.0 [56]. Geographic distances were taken as straight-line distances between localities.

A haplotype network was built using TCS 1.21 [57]; this network also provides an overall visual assessment of the haplotype diversity in each of the localities. In addition, potential barriers to gene-flow were identified using an interpolation-based graphic approach in the programme Alleles In Space [56]. To investigate the genetic clustering of individuals across the range of *Georychus*, clustering analyses were performed in BAPS version 6.0 [58, 59, 60] by employing both a normal clustering search (without geographic data) and a spatial clustering search (using the coordinates of sampled localities).

#### Comparison to *B*. *suillus*

Summary diversity indices (haplotype diversity and nucleotide diversity), population demography (Fu’s Fs) and population differentiation (pairwise Ф_ST_ values) were sourced from a previous study on *B*. *suillus* [4] for which the sampling scheme followed a similar broad spatial pattern across the Western Cape region. Estimates of Θ were also calculated for the *B*. *suillus* sequence datasets. For both *G*. *capensis* and *B*. *suillus*, comparative statistics were calculated separately for both the control region and cytochrome *b* datasets (data for *B*. *suillus* sourced from [4]) as well as for a combined dataset of these two fragments. To account for a possible bias in the spatial scale of sampling, the data from *Georychus* was compared to that of *B*. *suillus* across two spatial scales: 1) all sampled localities across the distribution of *G*. *capensis* (localities 1–15) and 2) only including localities across the Western Cape Province (localities 1–12). In addition, genetic structure within the two genera was only compared across their distributions in the Western Cape region. Statistical comparisons between values were performed using a non-parametric Mann-Whitney U test as implemented in IBM SPSS Statistics version 20.0.0 (International Business Machines Corporation 2011).

## Results

**Fig 3:**
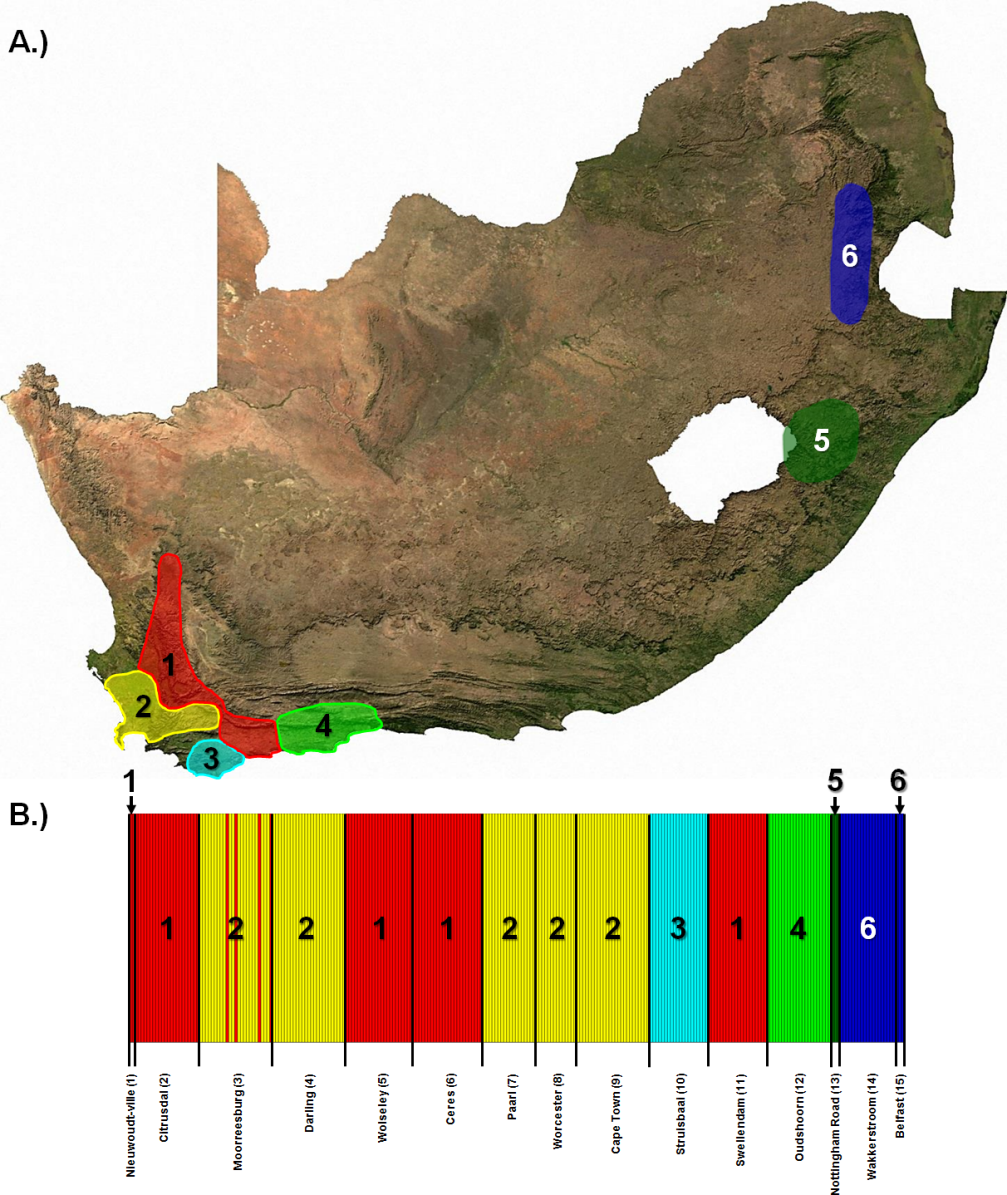
Genetic clusters in *G*. *capensis* across its distribution. A) The geographic spread of the different genetic clusters found within *G*. *capensis* with B) representing a breakdown of the populations constituting each cluster. The bars represent the haplotype of each individual with the colour indicating the membership of that individual to a particular genetic cluster. The colours in (B) correspond to the geographic position of the clusters in space (A).

When considering all 265 sampled *Georychus* individuals, 55 haplotypes were identified from the combined dataset (Table 1, Fig 2). With the exception of the Swellendam (locality 11) and Paarl (locality 7) localities, individual populations, as well as all populations combined, showed signs of negative population growth (Fu’s Fs values, Table 1). The trend of negative population growth was also retrieved when the data were partitioned into the separate genetic clusters over the distribution (Fig 3), with the exception of cluster 2 in the cytochrome *b* dataset which showed slight but significant positive population growth (Table 2). The genetic clusters over the sampled distribution (Fig 3) represent the number of genetically divergent groups based on the combined dataset. A genetic cluster therefore incorporates all individuals (populations) which are genetically similar, or are less divergent from one another than from individuals in another cluster. In addition to negative population growth, effective population sizes for *G*. *capensis* populations were relatively low (S1 Table) with six populations (localities 2, 3, 4, 5, 6 and 9; S1 Fig) having slightly higher Θ values compared to the other populations in all datasets.

**Table 1:** Genetic diversity within *G*. *capensis* populations.

Locality	Sample size (N)	Number of haplotypes	Haplotype diversity	Nucleotide diversity	Fu's Fs
Nieuwoudt-ville (1)	2	1	0.000 ± 0.000	0.000	a
Citrusdal (2)	22	3	0.554 ± 0.097	0.028	15.650***
Moorreesburg (3)	25	5	0.423 ± 0.119	0.015	13.958***
Darling (4)	25	9	0.793 ± 0.064	0.017	4.356*
Wolseley (5)	23	9	0.795 ± 0.076	0.034	5.264*
Ceres (6)	24	7	0.855 ± 0.033	0.028	9.064***
Paarl (7)	18	2	0.471 ± 0.082	0.000	1.215^n.s.^
Worcester (8)	14	2	0.264 ± 0.136	0.013	5.748*
Cape Town (9)	25	6	0.800 ± 0.049	0.031	8.132***
Struisbaai (10)	20	2	0.442 ± 0.088	0.022	23.371***
Swellendam (11)	20	2	0.190 ± 0.108	0.000	2.248^n.s.^
Oudshoorn (12)	22	1	0.000 ± 0.000	0.000	b
Nottingham Road (13)	3	2	0.667 ± 0.314	0.022	a
Wakkerstroom (14)	19	2	0.200 ± 0.112	0.006	16.331***
Belfast (15)	3	2	0.667 ± 0.314	0.010	a
**Total**	265	55	0.475 ± 0.106	0.015	41.468***

Genetic diversity for the sampled *G*. *capensis* populations (refer to Fig 1) based on the combined dataset. The number of specimens (N), number of haplotypes, haplotype diversity, nucleotide diversity and Fu’s F values is given for each population. For the Fu’s F values

n.s. = non-significant

* = p<0.05 and

*** = p<0.001.

An indication is given where the analysis could not be performed due to “a” too few samples from that population, or “b” all individuals within that population having the same haplotype.

**Table 2:** Demographic stability of *G*. *capensis* genetic clusters.

	Cytochrome *b*	Control Region	Combined
Cluster 1	-0.064n.s.	7.245***	8.809***
Cluster 2	-2.668*	7.507***	7.522***
Cluster 3	5.788*	21.717***	23.371***
Cluster 4	b	b	b
Cluster 5	a	a	a
Cluster 6	4.312*	15.066***	17.571***

Table showing the Fu’s Fs values for the various genetic clusters retrieved across the distribution of *G*. *capensis* (see Fig 3) and based on the separate (cytochrome *b* and control region) and combined datasets. For the Fu’s F values

n.s. = non-significant

* = p<0.05

*** = p<0.001.

An indication is given where the analysis could not be performed due to “a” too few samples from that cluster, and “b” all individuals within that cluster having the same haplotype.

**Fig 4:**
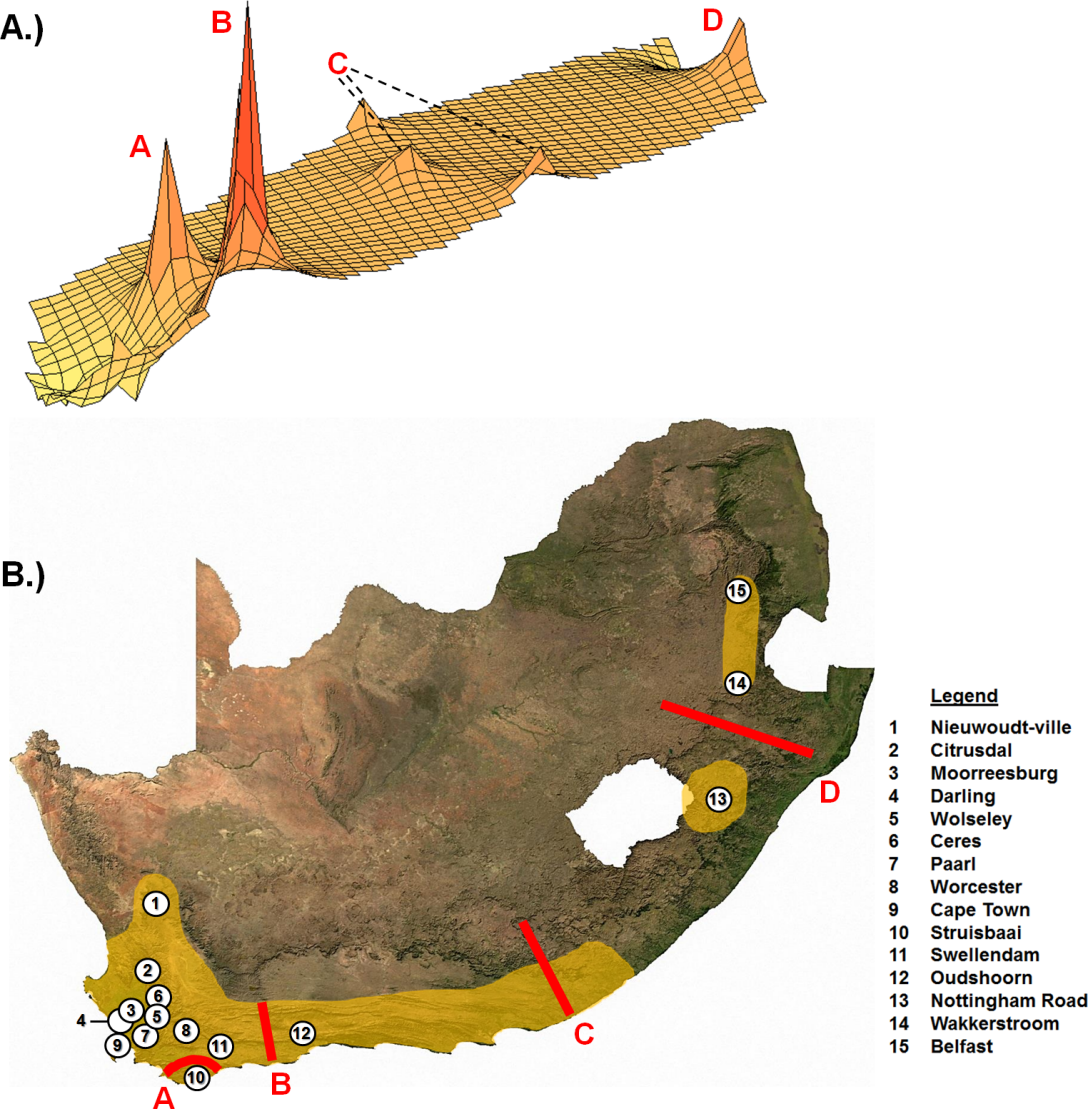
Barriers to gene-flow across the distribution of *G*. *capensis*. A) The genetic landscape (derived from the interpolation analysis in Alleles In Space) of *G*. *capensis* across the sampled distribution. Peaks on the landscape represent areas that are probable barriers to gene-flow (labelled A-D) with B) showing the location of these barriers on the distribution map of *G*. *capensis*.

There was significant and strong genetic structure across the range of *G*. *capensis* (Table 3; S3 Table), with every sampling locality presenting as a unique genetic entity (i.e., no haplotypes were shared between localities, see Fig 2). When all populations are considered together, 86% of the variation is accounted for by the between population component (Ф_ST_ = 0.863, df = 264, p = 0.000), with only 14% of the variation situated within populations. When three groups were specified in the AMOVA analysis (Western Cape, KwaZulu-Natal and Mpumalanga Provinces respectively), 47% of the variation was partitioned among these groups, with 44% of the variation among populations within each group, and only 8% of the variation partitioned within these populations. In the Western Cape region, a similar scenario was found with 83% of the variation among populations with only 16% of the variation accounted for by within-population variation (Ф_ST_ = 0.835, df = 239, p = 0.000). Although there was significant isolation-by-distance in the Western Cape region (r = 0.820, p = 0.001), several barriers to gene flow were detected across the range of *G*. *capensis* (Fig 4), contributing to the overall strong pattern of population structure.

**Table 3:** Genetic structure between *G*. *capensis* populations (combined dataset).

Locality	Nieuwoudt-ville	Citrusdal	Moorreesburg	Darling	Wolseley	Ceres	Paarl	Worcester	Cape Town	Struisbaai	Swellendam	Oudshoorn	Nottingham Road	Wakkerstroom	Belfast
Nieuwoudt-ville	-														
Citrusdal	0.612**	-													
Moorreesburg	0.771**	0.735***	-												
Darling	0.764**	0.577***	0.771***	-											
Wolseley	0.416*	0.196**	0.669***	0.550***	-										
Ceres	0.569**	0.331***	0.735***	0.561***	0.237***	-									
Paarl	0.995**	0.695***	0.857***	0.590***	0.605***	0.646***	-								
Worcester	0.772**	0.592***	0.774***	0.485***	0.512***	0.557***	0.589***	-							
Cape Town	0.645**	0.680***	0.377***	0.708***	0.630***	0.690***	0.754***	0.664***	-						
Struisbaai	0.824**	0.788***	0.861***	0.831***	0.752***	0.771***	0.885***	0.830***	0.813***	-					
Swellendam	0.994**	0.715***	0.809***	0.858***	0.650***	0.694***	0.993***	0.903***	0.715***	0.906***	-				
Oudshoorn	1.000**	0.948***	0.972***	0.967***	0.937***	0.949***	1.000***	0.983***	0.942***	0.965***	0.999***	-			
Nottingham Road	0.944^n.s.^	0.858***	0.925***	0.914***	0.840***	0.871***	0.989**	0.935**	0.856***	0.902***	0.987***	0.992***	-		
Wakkerstroom	0.974**	0.902***	0.939***	0.938***	0.886***	0.905***	0.983***	0.953***	0.893***	0.925***	0.981***	0.989***	0.947**	-	
Belfast	0.975^n.s.^	0.863***	0.928***	0.916***	0.844***	0.873***	0.994***	0.941**	0.859***	0.905***	0.993***	1.000***	0.881^n.s.^	0.879***	-

Pairwise ɸ_ST_ values between the sampled *G*. *capensis* populations based on the combined dataset.

n.s. = non-significant

* = p<0.05

** = p<0.01

*** = p<0.001.

*Georychus capensis* populations exhibited significantly lower haplotype diversity compared to *B*. *suillus* populations for all datasets (fragments singly or combined) and across both the spatial scales (Table 4). When comparing nucleotide diversity values, *G*. *capensis* exhibited significantly lower diversity compared to *B*. *suillus* (on both studied spatial scales) only for the cytochrome *b* dataset, with non-significant values for the control region and combined data (see Table 4), possibly suggesting negative population growth in *G*. *capensis*. Indeed, the Fu’s Fs values of *G*. *capensis* were significantly higher compared to that of *B*. *suillus* in all datasets and across both spatial scales, with exception of the values based on the cytochrome *b* datasets in the Western Cape region (Table 4). In addition, *G*. *capensis* exhibited significantly lower effective population sizes compared to *B*. *suillus* (on both spatial scales) based on all datasets (Table 4). *Georychus capensis* was spatially more structured compared with *B suillus* in the Western Cape region (cytochrome *b* results only, see Table 4).

**Table 4:** Statistical results for the comparisons between *G*. *capensis* and *B*. *suillus* genetic indices.

**Cytochrome *b***						
**All populations**	**Mann-Whitney U**	**N**_***Georcyhus***_	**N**_***Bathyergus***_	**p-value**	**Mean**_***Georcyhus***_ **± SD**	**Mean**_***Bathyergus***_ **± SD**
Haplotype diversity	30.000	15	10	**0.012**	0.411 ± 0.292	0.680 ± 0.235
Nucleotide diversity	37.500	15	10	**0.033**	0.001 ± 0.001	0.003 ± 0.002
Fu's FS	18.000	9	10	**0.027**	2.404 ± 2.781	-0.053 ± 1.089
Θ	16.000	12	10	**0.004**	0.001 ± 0.000	0.002 ± 0.002
**Western Cape**	**Mann-Whitney U**	**N**_***Georcyhus***_	**N**_***Bathyergus***_	**p-value**	**Mean**_***Georcyhus***_ **± SD**	**Mean**_***Bathyergus***_ **± SD**
Haplotype diversity	23.500	12	10	**0.016**	0.385 ± 0.304	0.680 ± 0.235
Nucleotide diversity	30.500	12	10	**0.046**	0.001 ± 0.001	0.003 ± 0.002
Fu's FS	18.000	8	10	0.051	2.052 ± 2.751	-0.053 ± 1.089
Θ	17.000	11	10	**0.005**	0.001 ± 0.000	0.002 ± 0.002
FST	1146.000	66	45	**0.042**	0.818 ± 0.226	0.764 ± 0.230
**Control Region**						
**All populations**	**Mann-Whitney U**	**N**_***Georcyhus***_	**N**_***Bathyergus***_	**p-value**	**Mean**_***Georcyhus***_ **± SD**	**Mean**_***Bathyergus***_ **± SD**
Haplotype diversity	20.500	15	10	**0.002**	0.438 ± 0.310	0.791 ± 0.170
Nucleotide diversity	55.000	15	10	0.267	0.033 ± 0.027	0.020 ± 0.010
Fu's FS	11.000	10	10	**0.003**	9.533 ± 5.781	2.474 ± 3.448
Θ	17.000	12	10	**0.005**	0.007 ± 0.010	0.034 ± 0.029
**Western Cape**	**Mann-Whitney U**	**N**_***Georcyhus***_	**N**_***Bathyergus***_	**p-value**	**Mean**_***Georcyhus***_ **± SD**	**Mean**_***Bathyergus***_ **± SD**
Haplotype diversity	16.500	12	10	**0.004**	0.420 ± 0.327	0.791 ± 0.170
Nucleotide diversity	43.000	12	10	0.262	0.034 ± 0.028	0.020 ± 0.010
Fu's FS	11.000	9	10	**0.006**	9.013 ± 5.879	2.474 ± 3.448
Θ	17.000	11	10	**0.007**	0.007 ± 0.011	0.034 ± 0.029
FST	1397.000	66	45	0.597	0.714 ± 0.197	0.719 ± 0.239
**Combined (cytochrome *b* and control region)**						
**All populations**	**Mann-Whitney U**	**N**_***Georcyhus***_	**N**_***Bathyergus***_	**p-value**	**Mean**_***Georcyhus***_ **± SD**	**Mean**_***Bathyergus***_ **± SD**
Haplotype diversity	14.000	15	10	**0.001**	0.475 ± 0.292	0.848 ± 0.174
Nucleotide diversity	55.000	15	10	0.265	0.015 ± 0.012	0.009 ± 0.004
Fu's FS	12.000	11	10	**0.002**	9.576 ± 6.917	1.328 ± 3.264
Θ	6.000	12	10	**0.000**	0.002 ± 0.003	0.018 ± 0.013
**Western Cape**	**Mann-Whitney U**	**N**_***Georcyhus***_	**N**_***Bathyergus***_	**p-value**	**Mean**_***Georcyhus***_ **± SD**	**Mean**_***Bathyergus***_ **± SD**
Haplotype diversity	12.000	12	10	**0.002**	0.466 ± 0.307	0.848 ± 0.174
Nucleotide diversity	43.000	12	10	0.260	0.016 ± 0.013	0.009 ± 0.004
Fu's FS	12.000	10	10	**0.004**	8.901 ± 6.898	1.328 ± 3.264
Θ	6.000	11	10	**0.001**	0.002 ± 0.003	0.018 ± 0.013
FST	1478.500	66	45	0.969	0.736 ± 0.189	0.713 ± 0.238

Summary of the compared results (*G*. *capensis* and *B*. *suillus*) for haplotype diversity, nucleotide diversity Fu’s Fs and Θ (a measure of effective population size) values. Comparisons are done for the DNA fragments separately (cytochrome *b* and control region) and combined. The two spatial scales investigated (the entire distribution of *G*. *capensis* and populations in the Western Cape only) are also included. Spatial structure comparisons (pairwise ɸ_ST_ values across this range for *G*. *capensis* and *B*. *suillus*) are also done for the Western Cape region. For each comparison, the Mann-Whitney U test statistic, number of *Georychus* values (N_*Georychus*_), number of *Bathyergus* values (N_*Bathyergus*_) and the p-value (significant values in bold) is given. In addition, the mean ± standard deviation (S.D) for *Georyhus* and *Bathyergus* are shown for the values in question.

## Discussion

Compared to *B*. *suillus*, *G*. *capensis* is characterized by low genetic diversity. This may be a result of several factors, including population declines at both local (single population) and regional (across the entire distribution) scales. Indeed, *G*. *capensis* populations show significant declines in population size compared to *B*. *suillus* irrespective of the spatial scale considered. The overall population decline in the Cape mole-rat is in line with the fossil record (*G*. *capensis* fossils are found in areas where they do not occur currently e.g., the Gauteng Province, parts of the KwaZulu Natal Province and Eastern Cape Province), which suggests that *Georychus* was once more widely distributed [61, 62, 63, 64]. Historical population contractions during the Quaternary [62, 64, 65] left populations in the Mpumalanga and KwaZulu-Natal Provinces as relicts [65]. Overall population declines may result from an increasingly fragmented range with the loss of suitable habitat, specifically adherence to constant mesic environments [19] resulting in extreme isolation of *G*. *capensis* populations. Isolation and fragmentation of this habitat may likely be attributed to several climatic oscillations which brought cooler and more arid climates during the Quaternary [65, 66, 67, 68, 69].

Genetic variation also varies non-randomly among population and species [70] and depends on several factors including the mating system of the species, population history and environmental heterogeneity [45]. Ecological factors have been shown to influence natural selection and therefore genetic diversity in fossorial taxa [70]; natural selection maintains higher levels of polymorphism in harsh and unpredictable environments in species such as *Spalax ehrenbergi* [71]. Conversely, environmental stability causes lower genetic variation in populations of *T*. *romana* [22]. *Georychus* is associated with mesic conditions and drainage systems (vleis or areas close to rivers; see [19]). Lower levels of genetic diversity in *G*. *capensis* population occupying stable environments might therefore not pose any risk to long-term persistence, largely because of the lack of environmental stressors.

Extreme isolation may also influence the genetic diversity of *G*. *capensis* populations. *Georychus* populations are characterized by strong genetic structure across the distribution. Habitat specificity and a low dispersal capability may be the main factors driving spatial genetic structure. Suitable habitat is disjunctly distributed across the species’ range, and populations are spatially highly fragmented. Indeed, it was noted that *G*. *capensis* is less widespread and populations much more isolated compared to other South African bathyergid counterparts such as *B*. *suillus* and *Cryptomys hottentotus* [19]. *Bathyergus suillus* occupies deep sandy soils [35]; this habitat type is abundant and largely continuous along the coastal margins of South Africa. In addition, *B*. *suillus* is able to utilize mesic areas (with deep soils), thereby resulting in a range overlap with *G*. *capensis*. Given its more generalist ecology, *B*. *suillus* exhibits much larger and aggregated populations with a continuous distribution along the coastal regions of the Western Cape (J.H. Visser, personal observation). *Bathyergus suillus* therefore maintains much larger female effective population sizes (when considering the maternally inherited markers used here; Table 4; S1 Table) and higher levels of gene-flow (also see [14]) across its distribution, thereby resulting in the maintenance of higher (mitochondrial) genetic diversity within this species compared to *G*. *capensis*.

The extreme isolation of *G*. *capensis* populations negates all chances of genetic exchange and therefore populations with small effective population sizes may consequently be subject to inbreeding and/or genetic drift (albeit testing and corroborating these factors will require the addition of variable nuclear data such as microsatellites or SNPs). Small effective population sizes are evident in all *G*. *capensis* populations (S1 Table). The six populations exhibiting slightly higher effective population sizes (S1 Fig) represent specimens sampled across larger and disparate areas (>2km apart). These populations are all located in the south-western Cape area and are the only localities where *G*. *capensis* individuals were distributed across larger areas (>1km^2^), therefore representing larger numbers of unrelated mating individuals (females) and higher levels of genetic diversity (Table 1). Effective population sizes are also affected by unequal sex ratios [45]. Visser *et al*. [19] reported skewed sex ratios in most of the *G*. *capensis* sampled populations. It is possible that *G*. *capensis* populations have become inbred over time and/or were subject to bottlenecks, thereby decreasing the genetic diversity of founder populations. In addition, genetic drift seems to have played a major role in structuring *G*. *capensis* populations. There are no shared haplotypes between populations. Although based on both mitochondrial and nuclear markers, a loss of genetic variation and concomitant increase in genetic differentiation through genetic drift, inbreeding and bottlenecks, has been demonstrated on a temporal scale of 10 years in *Geomys bursarius* (see [9]). This temporal differentiation was found to be of the same magnitude as spatial differentiation in fossorial rodents [9]. In addition, genetic structure has been shown to result from bottlenecks and small effective population sizes in two geographically restricted populations (12 km apart) in *Ctenomys* [10].

Given the high genetic structure and geographic isolation among *G*. *capensis* populations, it is no surprise that significant isolation-by-distance was detected for this species in the Western Cape. Geographical distance may represent an insurmountable barrier to gene-flow for this poorly dispersing habitat specialist species.

In addition, long-standing geographic barriers such as major rivers and mountain ranges (similarly to *B*. *suillus*; [4]), elevation and biogeographic differences and different dispersal routes also play a major role in structuring genetic diversity in *G*. *capensis*. The six genetic clusters across the distribution of *G*. *capensis*, especially in the Western Cape, cannot be explained by isolation-by-distance alone.

The action of mountains on gene-flow in *G*. *capensis* seems to be that of a physical barrier having a channelling effect; in *B*. *suillus*, mountains form a barrier between populations [4]. Within the Western Cape, Clusters 1 and 2 (the Nieuwoudt-ville/Citrusdal/Wolseley/Ceres/Swellendam cluster and the Moorreesburg/Darling/Paarl/Cape Town cluster, respectively) is likely a result of different dispersal routes. Cluster 2 is located on the low-lying areas of the south-western Cape and is separated from Cluster 1 by the Olifants River-, Boland- and Hottentots Holland Mountains. Cluster 1 is found inland between the Olifants River-, Boland- and Hottentots Holland Mountains and the Cederberg-, Skurweberg-, Hex River- and Langeberg Mountains. It is possible that Cluster 1 spread along the Breede River Valley and followed the natural north-south valleys between the mountain ranges while spreading northward. The two clusters therefore appear to have resulted from two different, but relatively recent dispersal routes. Three individuals from the Moorreesburg locality originate from Cluster 1 and likely represent an isolated, unidirectional gene-flow event from one of the geographically close localities to Cluster 2 (Citrusdal/Wolseley areas, or areas in between) through one of the natural valleys that connect the coastal plain and the Berg River Valley.

The isolation of Cluster 3 (the Struisbaai population) may be a result of isolation through marine fluctuations in sea-level which covered the Agulhas Plain (barrier A on Fig 4; [72, 73, 74]). Indeed, sea-level fluctuations structure fossorial populations through fragmentation and isolation during transgressions [22, 24]. In addition, the action of rivers drives the isolation of fossorial populations ([10, 71, 75], but see [24]). It is therefore also likely that Cluster 3 was isolated from surrounding populations by the geographic barriers of the Breede River and Cape Fold Mountains (see [4] for a similar pattern in *B*. *suillus*). In addition, the action of the Breede River and Gourits River (barrier B on Fig 4), both of which was found as significant geographic barriers to gene-flow in *B*. *suillus* [4], may also drive the isolation of Cluster 4 (the Oudshoorn population). Conversely, Cluster 4 was found nestled within an enclosed montane valley, which could have contributed to its isolation.

Clusters 5 and 6 represent relict populations [65] in the Mpumalanga and KwaZulu-Natal Provinces (with possibly no intermediate populations). These clusters (populations) are separated by large geographic distances (>500km) from each other (barrier D on Fig 4) and from those in the Western Cape (barrier C on Fig 4). Although such large scale isolation has likely persisted for a protracted period of time (due to population declines; see above), several possible long-standing barriers also exist in the form of elevation and biogeographic differences across these regions [19]. These barriers developed through geological and climatic changes in the Miocene and Pliocene/Pleistocene, including pronounced tectonic uplift along the margins of the Great Escarpment [76, 77, 78], sea-level fluctuations which inundated and exposed regions of the coastal shelf [72, 74, 79, 80] and climatic conditions which shifted rapidly from warm and tropical towards a cooler, drier and more seasonal [78, 81, 82]. In addition, these changes promoted the spread of grasslands in the interior [76] and fragmented the woodland savannah vegetation that existed across this region [66, 83, 84].

## Conclusion

*Georychus capensis* is a subterranean species, which exhibits low genetic diversity relative to its concomitantly distributed sister genus, *Bathyergus*. This species shows strict adherence to certain ecological variables [19]; the spatial heterogeneity in the distribution of such suitable habitat results in geographically discrete and isolated populations. Possibly, because of these considerations, the extreme isolation of *G*. *capensis* populations leads to local population declines and low effective population sizes—inbreeding and bottlenecks may hence reduce genetic diversity. Also linked to this long term isolation is genetic drift which has led to each population constituting genetically unique entities. It therefore appears that the habitat specificity of the species and life-history along with habitat heterogeneity and geographic distance leads to isolation and differentiation, thereby resulting in high genetic structure between *G*. *capensis* populations.

In addition, the historical and contemporary actions of geographic barriers (mountains and rivers), sea-level fluctuations and elevation along with biogeographic differences act in concert with contractions of the distribution of *G*. *capensis* to produce long-term isolation of populations. As such, this has resulted in several genetically discrete clusters across the distribution of *G*. *capensis*. Specifically, mountains act as geographic barriers, either through channelling dispersal events or through isolating *G*. *capensis* populations in the Western Cape. In addition, major rivers and possibly historical changes in sea-level have caused the long-term isolation of populations in the coastal areas of the Western Cape region. The relict and disjunct Mpumalanga and KwaZulu-Natal *G*. *capensis* populations, which are also separated from those in the Western Cape by large geographic distances and elevation- and biogeographic differences, may have been isolated and diverging for a protracted time.

Speciation has been suggested to be linked to population structure and geographic isolation in various fossorial/subterranean taxa (e.g., [10, 85, 86, 87, 88, 89, 90]). Given the high degree of genetic isolation and differentiation among *G*. *capensis* populations along with possible adaptive differences across the distribution (see [19]), it is entirely possible that the monotypic genus *Georychus* is in need of a taxonomic revision as it may represent a species complex. In addition, conservation initiatives should take cognisance of the isolation and divergence of *G*. *capensis* population, both locally and on a distribution-wide scale, and the low level of genetic diversity within these isolates.

## Supporting information

S1 Table Θ values for *G*. *capensis* and *B*. *suillus* populations. Θ values (a measure of effective population size for the sampled *Georychus* populations as well as the *B*. *suillus* populations in Visser *et al*. [4]. For each population, Θ values are shown for the cytochrome *b* and control region datasets separately, as well as for the combined dataset of these markers. An “a” indicates where reliable results could not be obtained due to too few samples from that population.
(DOCX)

S2 Table Genetic diversity of *G*. *capensis* populations. Genetic diversity of the sampled *G*. *capensis* populations showing the haplotype diversity, nucleotide diversity and Fu’s F values in each population for the cytochrome *b*/control region datasets. For the Fu’s F values n.s. = non-significant,* = p<0.05, ** = p<0.01, *** = p<0.001. An indication is given where the analysis could not be performed due to “a” too few samples from that population and “b” all individuals within that population having the same haplotype.
(DOCX)

S3 Table Genetic structure between *G*. *capensis* populations (cytochrome *b* and control region separately). Pairwise ɸ_ST_ values between the sampled *G*. *capensis* populations with values based on cytochrome *b* below the diagonal and values based on the control region above the diagonal. n.s. = non-significant,* = p<0.05, ** = p<0.01, *** = p<0.001.
(DOCX)

S4 Table Genetic diversity of *B*. *suillus* populations. Genetic diversity of the *B*. *suillus* populations in [4] populations showing the haplotype diversity, nucleotide diversity and Fu’s F values in each population for the cytochrome *b*/control region datasets. For the Fu’s F values n.s. = non-significant,* = p<0.05, ** = p<0.01, *** = p<0.001.
(DOCX)

S5 Table Genetic structure between *B*. *suillus* populations (cytochrome *b* and control region separately). Pairwise ɸ_ST_ values between the sampled *B*. *suillus* populations in [4] with values based on cytochrome *b* below the diagonal and values based on the control region above the diagonal. n.s. = non-significant,* = p<0.05, ** = p<0.01, *** = p<0.001.
(DOCX)

S1 Fig Θ values for the *G*. *capensis* populations (for the cytochrome *b* and control region markers separately and combined). Plot showing the Θ values for the sampled *G*. *capensis* populations based on the A) cytochrome *b* dataset, B) control region dataset and C) combined (cytochrome *b* control region) dataset.
(TIF)

## References

[1] Jansen van Vuuren B, Robinson TJ. Genetic population structure in the yellow mongoose, *Cynictis penicillata*. Mol Ecol. 1997;6: 1147–1153. 9421919 10.1046/j.1365-294x.1997.00290.x

[2] Mortimer E, Jansen van Vuuren B, Meiklejohn KI, Chown SL. Phylogeography of a mite, *Halozetes fulvus*, reflects the landscape history of a young volcanic island in the sub-Antarctic. Biol J Linn Soc. 2012;105: 131–145.

[3] Du Toit N, Jansen van Vuuren B, Matthee S, Matthee CA. Biogeography and host-related factors trump parasite life history: Limited congruence among the genetic structures of specific ectoparasitic lice and their rodent hosts. Mol Ecol. 2013;22: 5185–5204. doi: 10.1111/mec.12459 24010927 10.1111/mec.12459

[4] Visser JH, Bennett NC, Jansen van Vuuren B. Local and regional scale genetic variation in the Cape dune mole-rat, *Bathyergus suillus*. PLoS ONE. 2014;9(9):e107226 doi: 10.1371/journal.pone.0107226 25229558 10.1371/journal.pone.0107226 PMC4167993

[5] Nevo E. Mammalian evolution underground. The ecological-genetic-phenetic interfaces. Acta Theriol. 1995;3: 9–31.

[6] Miller RS. Ecology and distribution of pocket gophers (Geomyidae) in Colorado. Ecology. 1964;45: 256–272.

[7] Nevo E. Adaptive convergence and divergence of subterranean mammals. Annu Rev Ecol Syst. 1979;10: 269–308.

[8] Davies KC, Jarvis JUM. The burrow systems and burrowing dynamics of the mole-rats *Bathyergus suillus* and *Cryptomys hottentotus* in the fynbos of the south-western Cape, South Africa. J Zool (Lond). 1986;209: 125–147.

[9] Zimmerman EG. Temporal genetic variation in a population of the pocket gopher, *Geomys bursarius*. Genetica. 1988;76: 153–159.

[10] Mirol P, Giménez MD, Searle JB, Bidau CJ, Faulkes CG. Population and species boundaries in the South American subterranean rodent *Ctenomys* in a dynamic environment. Biol J Linn Soc. 2010;100: 368–383.

[11] Tang L, Wang L, Cai Z, Zhang T, Ci H, Lin G, et al Allopatric divergence and phylogeographic structure of the plateau zokor (*Eospalax baileyi*), a fossorial rodent endemic to the Qinghai–Tibetan Plateau. J Biogeogr. 2010;37: 657–668.

[12] Welborn SR, Light JE. Population genetic structure of the Baird’s pocket gopher, *Geomys breviceps*, in eastern Texas. West N Am Naturalist. 2014;74: 325–334.

[13] Šklíba JS, Šumbera R, Chitaukali WN, Burda H. Home-range dynamics in a solitary subterranean rodent. Ethology. 2009;115: 217–226.

[14] Bray TC, Jansen van Rensburg A, Bennett NC. Overground versus underground: a genetic insight into dispersal and abundance of the Cape dune mole-rat. Biol J of the Linn Soc. 2013;110: 890–897.

[15] Cook JA, Lessa EP. Are rates of diversification in subterranean South American tuco-tucos (genus *Ctenomys*, Rodentia: Octodontidae) unusually high? Evolution. 1998;52: 1521–1527. doi: 10.1111/j.1558-5646.1998.tb02035.x 28565377 10.1111/j.1558-5646.1998.tb02035.x

[16] Jarvis JUM, Sherman PW. Mammalian species No. 706: *Heterocephalus glaber*. Am Soc Mammal. 2002; 1–9.

[17] Wlasiuk G, Garza JC, Lessa EP. Genetic and geographic differentiation in the Rio Negro tuco-tuco (*Ctenomys rionegrensis*): Inferring the roles of migration and drift from multiple genetic markers. Evolution. 2003;57: 913–926. 12778560 10.1111/j.0014-3820.2003.tb00302.x

[18] Mapelli FJ, Kittlein MJ. Influence of patch and landscape characteristics on the distribution of the subterranean rodent *Ctenomys porteousi*. Landscape Ecol. 2009;24: 723–733.

[19] Visser JH, Bennett NC, Jansen van Vuuren B. Distributional range, ecology and mating system of the Cape mole-rat, *Georychus capensis* family Bathyergidae. Can J Zool. 2017;95: 713–726.

[20] Faulkes CG, Verheyen E, Verheyen W, Jarvis JUM, Bennett NC. Phylogeographical patterns of genetic divergence and speciation in African mole-rats (Family: *Bathyergidae*). Mol Ecol. 2004;13: 613–629. 14871365 10.1046/j.1365-294x.2004.02099.x

[21] Šumbera R, Šklíba J, Elichová M, Chitaukali WN, Burda H. Natural history and burrow system architecture of the silvery mole-rat from Brachystegia woodland. J Zool. 2008;274: 77–84.

[22] Canestrelli D, Aloise G, Cecchetti S, Nascetti G. Birth of a hotspot of intraspecific genetic diversity: notes from the underground. Mol Ecol. 2010;19: 5432–5451. doi: 10.1111/j.1365-294X.2010.04900.x 21059127 10.1111/j.1365-294X.2010.04900.x

[23] Mapelli FJ, Mora MS, Mirol PM, Kittlein MJ. Effects of Quaternary climatic changes on the phylogeography and historical demography of the subterranean rodent *Ctenomys porteousi*. J Zool. 2012;286: 48–57.

[24] Roratto PA, Fernandes FA, de Freitas TRO. Phylogeography of the subterranean rodent *Ctenomys torquatus*: an evaluation of the riverine barrier hypothesis. J Biogeogr. 2014;42: 1–12.

[25] Pallas PS. Novae species Quadrupedum e Glirium ordine, cum illustrationibus varies complurium ex hoc ordine Animalium. Volumes 1 and 2 Germany: Walther; 1778.

[26] Taylor PJ, Jarvis JUM, Crowe TM. Age determination in the Cape mole-rat *Georychus capensis*. S Afr J Zool. 1985;20: 261–267.

[27] Bennett NC, Jarvis JUM. The reproductive biology of the Cape mole-rat, *Georychus capensis* (Rodentia: Bathyergidae). J Zool (Lond). 1988;214: 95–106.

[28] Narins PM, Reichman OJ, Jarvis JUM, Lewis ER. Seismic signal transmission between burrows of the Cape mole-rat, *Georychus capensis*. J Comp Physiol. 1992;170: 13–21. 1573567 10.1007/BF00190397

[29] Du Toit JT, Jarvis JUM, Louw GN. Nutrition and burrowing energetics of the Cape mole-rat *Georychus capensis*. Oecologia. 1985;66: 81–87. doi: 10.1007/BF00378556 28310816 10.1007/BF00378556

[30] Bennett NC. The trend towards sociality in three species of southern African mole-rats Bathyergidae: causes and consequences. Ph.D. thesis, University of Cape Town; 1988. Available from: https://open.uct.ac.za/bitstream/handle/11427/8413/thesis_sci_1988_bennett_nc.pdf?sequence=1.

[31] Bennett NC, Maree S, Faulkes CG. Mammalian Species: *Georychus capensis*. Am Soc Mammal. 2006;799: 1–4.

[32] De Graaff G. The rodents of Southern Africa. South Africa: Butterworth; 1981.

[33] Nanni RF. The interaction of mole-rats (*Georychus capensis* and *Cryptomys hottentotus*) in the Nottingham road region of Natal. Unpublished M.Sc.thesis, University of Natal; 1988. Available from: http://nrfnexus.nrf.ac.za/handle/20.500.11892/84452.

[34] Bronner GN. New distribution records for four mammal species, with notes on their taxonomy and ecology. Koedoe. 1990;33: 1–7.

[35] Skinner JD, Chimimba CT. The mammals of the Southern African subregion Cambridge: Cambridge University Press; 2005.

[36] Nevo E, Capanna E, Corti M, Jarvis JUM, Hickman GC. Karyotype differentiation in the endemic subterranean mole rats of South Africa (Rodentia, Bathyergidae). Z Saugetierkd. 1986;51: 36–49.

[37] Deuve JL, Bennett NC, Britton-Davidian J, Robinson TJ. Chromosomal phylogeny and evolution of the African mole-rats (Bathyergidae). Chromosome Res. 2008;16: 57–74. doi: 10.1007/s10577-007-1200-8 18293105 10.1007/s10577-007-1200-8

[38] Honeycutt RL, Edwards SV, Nelson K, Nevo E. Mitochondrial DNA variation and the phylogeny of African mole rats (Rodentia: Bathyergidae). Syst Zool. 1987;36: 280–292.

[39] Nevo E, Ben-Shlomo R, Belles A, Jarvis JUM, Hickman GC. Allozyme differentiation and systematics of the endemic subterranean mole rats of South Africa. Biochem Syst Ecol. 1987;15: 489–502.

[40] Honeycutt RL, Allard MW, Edwards SV, Schlitter DA. Systematics and evolution of the family Bathyergidae In: Sherman PW, Jarvis JUM, Alexander R, editors. The biology of the naked mole-rat. Princeton: Princeton University Press; 1991 pp. 45–65.

[41] Ingram CM, Burda H, Honeycutt RL. Molecular phylogenetics and taxonomy of the African mole-rats, genus *Cryptomys* and the new genus *Coetomys* (Gray, 1864). Mol Phylogenet Evol. 2004;31: 997–1014. doi: 10.1016/j.ympev.2003.11.004 15120397 10.1016/j.ympev.2003.11.004

[42] Maree S, Faulkes C. Georychus capensis. The IUCN Red List of Threatened Species 2008;e.T9077A12955652. Available from: http://dx.doi.org/10.2305/IUCN.UK.2008.RLTS.T9077A12955652.en.

[43] McNeely JA, Miller KR, Reid WV, Mittermeier RA, Werner TB. Conserving the world’s biological diversity Washington DC: World Conservation Union, World Resources Institute, Conservation International, World Wildlife Fund-US, and the World Bank; 1990.

[44] Briscoe DA, Malpica JM, Robertson A, Smith GJ, Frankham R, Banks RG et al Rapid loss of genetic variation in large captive populations of *Drosophila* flies: Implications for the genetic management of captive populations. Conserv Biol. 1992;6: 416–425.

[45] Booy G, Hendriks RJJ, Smulders MJM, Van Groenendael JM, Vosman B. Genetic diversity and the survival of populations. Plant Biol. 2000;2: 379–395.

[46] Reed DH, Frankham R. Correlation between fitness and genetic diversity. Conserv Biol. 2003;17: 230–237.

[47] Garner A, Rachlow JL, Hicks JF. Patterns of genetic diversity and its loss in mammalian populations. 2004;DOI: 10.1111/j.1523-1739.2005.00105.x

[48] Kocher TD, Thomas WK, Meyer A, Edwards SV, Paabo S, Villablanca X, et al Dynamics of mitochondrial DNA evolution in animals: Amplification and sequencing with conserved primers. P Natl Acad Sci USA. 1989;86: 6196–6200. 10.1073/pnas.86.16.6196 PMC297804 2762322

[49] Irwin DM, Kocher TD, Wilson AC. Evolution of the cytochrome *b* gene of mammals. J Mol Evol. 1991;32: 128–144. 1901092 10.1007/BF02515385

[50] Patterson BD, Velazco PM. Phylogeny of the rodent genus *Isothrix* (Hystricognathi, Echimyidae) and its diversification in Amazonia and the eastern Andes. J Mammal Evol. 2008;15: 181–201.

[51] Smit HA, Robinson TJ, Watson J, Jansen van Vuuren B. A new species of elephant-shrew (Afrotheria: Macroscelidea: *Elephantulus*) from South Africa. J Mammal. 2008;89: 1257–1269.

[52] Jansen van Vuuren B, Chown SL. Genetic evidence confirms the origin of the house mouse on sub-Antarctic Marion Island. Polar Biol. 2007;30: 327–332.

[53] Excoffier L, Lischer HE. Arlequin suite ver 3.5: a new series of programs to perform population genetics analyses under Linux and Windows. Mol Ecol Resour. 2010;10: 564–567. doi: 10.1111/j.1755-0998.2010.02847.x 21565059 10.1111/j.1755-0998.2010.02847.x

[54] Librado P, Rozas J. DnaSP v5: A software for comprehensive analysis of DNA polymorphism data. Bioinformatics. 2009;25: 1451–1452. doi: 10.1093/bioinformatics/btp187 19346325 10.1093/bioinformatics/btp187

[55] Beerli P. How to use migrate or why are Markov Chain Monte Carlo programs difficult to use? In: Bertorelle G, Bruford MW, Hauffe HC, Rizzoli A, Vernesi C, editors. Population Genetics for Animal Conservation, Volume 17 of Conservation Biology. Cambridge, Cambridge University Press; 2009 pp. 42–79.

[56] Miller MP. Alleles In Space (AIS): Computer software for the joint analysis of interindividual spatial and genetic information. J Hered. 2005;96: 722–724. doi: 10.1093/jhered/esi119 16251514 10.1093/jhered/esi119

[57] Clement MD, Posada D, Crandall KA. TCS: A computer program to estimate gene genealogies. Mol Ecol. 2000;9: 1657–1660. 11050560 10.1046/j.1365-294x.2000.01020.x

[58] Corander J, Tang J. Bayesian analysis of population structure based on linked molecular information. Math Biosci. 2007;205: 19–31. doi: 10.1016/j.mbs.2006.09.015 17087977 10.1016/j.mbs.2006.09.015

[59] Corander J, Marttinen P, Sirén J, Tang J. Enhanced Bayesian modelling in BAPS software for learning genetic structures of populations. BMC Bioinformatics. 2008a;9: 539. 19087322 10.1186/1471-2105-9-539 PMC2629778

[60] Corander J, Sirén J, Arjas E. Bayesian spatial modelling of genetic population structure. Computation Stat. 2008b;23: 111–129.

[61] Hendey QB. Quaternary vertebrate fossil sites in the south-western Cape Province. S Afr Archaeol Bull. 1969;24: 96–105.

[62] Klein RG. A provisional statement on terminal Pleistocene mammalian extinctions in the Cape Biotic Zone (southern Cape Province, South Africa). S Afr Archaeol Bull. 1974;2: 39–45.

[63] Avery DM. Late Quarternary incidence of some micromammalian species in Natal. Durban Mus Novit. 1991;16: 1–16.

[64] Avery DM. An assessment of the lower Pleistocene micro mammalian fauna from Swartkrans members 1–3, Gauteng, South Africa. Geobios. 1998;31: 393–414.

[65] Avery DM. Notes on the systematics of micromammals from Sterkfontein, Gauteng, South Africa. Palaeontol Afr. 2000;36: 83–90.

[66] Van Zinderen Bakker EM, Mercer JH. Major late Cainozoic climatic events and palaeoenvironmental changes in Africa viewed in a worldwide context. Palaeogeogr Palaeoeccl. 1986;56: 217–235.

[67] Lawes MJ. The distribution of the samango monkey (*Cercopithecus mitis erythrarchus* Peters, 1852 and *Cercopithecus mitis labiatus* I. Geoffroy, 1843) and the forest history in southern Africa. J Biogeogr. 1990;17: 669–680.

[68] Demenocal PB. African climate change and faunal evolution during the Pliocene-Pleistocene. Earth Planet. Adv Sci Lett. 2004;220: 3–24.

[69] Lawes MJ, Eeley HAC, Findlay NJ, Forbes D. Resilient forest faunal communities in South Africa: a legacy of palaeoclimatic change and extinction filtering? J Biogeogr. 2007;34: 1246–1264.

[70] Nevo E, Kirzhner V, Beiles A, Korol A. Selection versus random drift: long-term polymorphism persistence in small populations (evidence and modelling). Philos T R Soc Lond. 1997;352: 381–389.

[71] Karanth KP, Avivi A, Beharav A, Nevo E. Microsatellite diversity in populations of blind subterranean mole rats (*Spalax ehrenbergi* superspecies) in Israel: speciation and adaptation. Biol J Linn Soc. 2004;83: 229–241.

[72] Van Andel TH. Late Pleistocene sea levels and the human exploitation of the shore and shelf of southern South Africa. J Field Archaeol. 1989;16: 133–155.

[73] Lambeck K. Sea-level change through the last glacial cycle: geophysical, glaciological geophysical, glaciological and palaeogeographic consequences. C R Geosci. 2004;336: 677–689.

[74] Compton JS. Pleistocene sea-level fluctuations and human evolution on the southern coastal plain of South Africa. Quaternary Sci Rev. 2011;30: 506–527.

[75] Mora MS, Mapelli FJ, López A, Fernández MJG, Mirol PM, Kittlein MJ. Population genetic structure and historical dispersal patterns in the subterranean rodent *Ctenomys “chasiquensis”* from the southeastern Pampas region, Argentina. Mamm Biol. 2016; Available at: http://dx.doi.org/10.1016/j.mambio.2016.02.008.

[76] Partridge TC, Maud RR. Macro-scale geomorphic evolution of southern Africa In: Partridge TC, Maud RR, editors. The Cenozoic of Southern Africa. New York: Oxford University Press; 2000 pp. 3–18.

[77] Sepulchre P, Ramstein G, Fluteau F, Schuster M, Tiercelin J, Brunet M. Tectonic uplift and eastern Africa aridification. Science 2006;313: 1419–1423. doi: 10.1126/science.1129158 16960002 10.1126/science.1129158

[78] Cowling RM., Procheş S, Partridge TC. Explaining the uniqueness of the Cape flora: Incorporating geomorphic evolution as a factor for explaining its diversification. Mol Phylogenet Evol. 2009;51: 64–74. doi: 10.1016/j.ympev.2008.05.034 18691908 10.1016/j.ympev.2008.05.034

[79] Deacon HJ. An introduction to the fynbos region, time scales and palaeoenvironments In: Deacon HJ, Hendey QB, Lambrechts NJJ, editors. Fynbos palaeoecology: a preliminary synthesis. Pretoria: CSIR; 1983 pp. 1–20.

[80] Hendey QB. Cenozoic geology and palaeogeography of the fynbos region In: Deacon HJ, Hendey QB, Lambrechts NJJ, editors. Fynbos palaeoecology: a preliminary synthesis. Pretoria: CSIR; 1983 pp. 35–60.

[81] Partridge TC. Evolution of landscapes In: Cowling RM, Richardson DM, Pierce SM, editors. Vegetation in southern Africa. Cambridge, Cambridge University Press; 1997 pp. 5–20.

[82] Linder HP. The radiation of the Cape flora, southern Africa. Biol Rev. 2003:78: 597–638. 14700393 10.1017/s1464793103006171

[83] Coetzee JA. Climatic and biological changes in south-western Africa during the Late Cainozoic In: Coetzee JA, van Zinderen Bakker EM, editors. Palaeoecology of Africa, Vol. 10 Rotterdam, Balkema; 1978 pp. 13–29.

[84] Coetzee JA. Intimations on the Tertiary vegetation of southern Africa. Bothalia 1983;14: 345–354.

[85] Thaeler CS. Karyotypes of sixteen populations of the *Thomomys talpoides* complex of pocket gophers (Rodentia: Geomyidae). Chromosoma. 1968;25: 172–183. 5709394 10.1007/BF00327176

[86] Nevo E, Ben-Shlomo R, Beiles A, Hart CP, Ruddle FH. Homeobox DNA polymorphisms (RFLPs) in subterranean mammals of the *Spalax ehrenbergi* superspecies in Israel: Patterns, correlates, and evolutionary significance. J Exp Zool. 1992;263: 430–441.

[87] Nevo E. Mosaic evolution of subterranean mammals: regression, progression and global convergence Oxford: Oxford University Press; 1999.

[88] Nevo E, Beiles A, Korol AB, Ronin YI, Pavlicek T, Hamilton W. Extraordinary multilocus genetic organization in mole crickets, Gryllotalpidae. Evolution. 2000;54: 586–605. 10937235 10.1111/j.0014-3820.2000.tb00061.x

[89] Van Daele PAAG, Faulkes CG, Verheyen E, Adriaens D. African mole-rats (Bathyergidae): a complex radiation in Afrotropical soils In: Begall S, Burda H, Schleich CE, editors. Subterranean rodents: news from underground. Heidelberg: Springer-Verlag; 2007 pp. 357–373.

[90] Kryštufek B, Ivanitskaya E, Arslan A, Arslan E, Bužan EV. Evolutionary history of mole rats (genus *Nannospalax*) inferred from mitochondrial cytochrome *b* sequence. Biol J Linn Soc. 2012;105: 446–455.

