# Supplementary material for: Spatial genetic diversity in the Cape mole-rat, Georychus capensis: Extreme isolation of populations in a subterranean environment
Source: PLoS One. 2018 Mar 15;13(3):e0194165. doi: 10.1371/journal.pone.0194165 (PMC5854370; doi:10.1371/journal.pone.0194165)
Supplement: S1 Table — Θ values (a measure of effective population size for the sampled Georychus populations as well as the B. suillus populations in Visser et al. [4]. For each population, Θ values are shown for the cytochrome b and control region datasets separately, as well as for the combined dataset of these markers. An “a” indicates where reliable results could not be obtained due to too few samples from that population. (DOCX) [file pone.0194165.s001.docx]

**S1 Table Θ values for *G. capensis* and *B. suillus* populations** Θ values (a measure of effective population size for the sampled *Georychus* populations as well as the *B. suillus* populations in Visser *et al*. [4]. For each population, Θ values are shown for the cytochrome *b* and control region datasets separately, as well as for the combined dataset of these markers. An “a” indicates where reliable results could not be obtained due to too few samples from that population.

| ***Georychus*** | **Cytochrome *b*** | **Control region** | **Combined (cytochrome *b* and control region)** |
| --- | --- | --- | --- |
| Nieuwoudt-ville (1) | a | a | a |
| Citrusdal (2) | 0.001 | 0.003 | 0.001 |
| Moorreesburg (3) | 0.001 | 0.002 | 0.002 |
| Darling (4) | 0.001 | 0.012 | 0.006 |
| Wolseley (5) | 0.002 | 0.037 | 0.010 |
| Ceres (6) | 0.001 | 0.007 | 0.002 |
| Paarl (7) | 0.000 | 0.001 | 0.000 |
| Worcester (8) | 0.000 | 0.002 | 0.001 |
| Cape Town (9) | 0.001 | 0.007 | 0.003 |
| Struisbaai (10) | 0.000 | 0.005 | 0.001 |
| Swellendam (11) | 0.000 | 0.001 | 0.001 |
| Oudshoorn (12) | 0.000 | 0.001 | 0.001 |
| Nottingham Road (13) | a | a | a |
| Wakkerstroom (14) | 0.000 | 0.001 | 0.001 |
| Belfast (15) | a | a | a |
| ***Bathyergus*** | **Cytochrome *b*** | **Control region** | **Combined (cytochrome *b* and control region)** |
| Sterkfontein | 0.002 | 0.065 | 0.018 |
| Vredenburg | 0.003 | 0.067 | 0.042 |
| Redelinghuys | 0.002 | 0.006 | 0.004 |
| Dwarskersbos | 0.003 | 0.058 | 0.029 |
| Piketberg | 0.000 | 0.005 | 0.004 |
| CapeTown | 0.006 | 0.077 | 0.030 |
| Stanford | 0.002 | 0.022 | 0.009 |
| Struisbaai | 0.001 | 0.020 | 0.010 |
| Riversdal | 0.001 | 0.018 | 0.009 |
| Sedgefield | 0.002 | 0.004 | 0.027 |
