# Supplementary material for: Spatial genetic diversity in the Cape mole-rat, Georychus capensis: Extreme isolation of populations in a subterranean environment
Source: PLoS One. 2018 Mar 15;13(3):e0194165. doi: 10.1371/journal.pone.0194165 (PMC5854370; doi:10.1371/journal.pone.0194165)
Supplement: S2 Table — Genetic diversity of the sampled G. capensis populations showing the haplotype diversity, nucleotide diversity and Fu’s F values in each population for the cytochrome b/control region datasets. For the Fu’s F values n.s. = non-significant,* = p<0.05, ** = p<0.01, *** = p<0.001. An indication is given where the analysis could not be performed due to “a” too few samples from that population and “b” all individuals within that population having the same haplotype. (DOCX) [file pone.0194165.s002.docx]

**S2 Table Genetic diversity of *G. capensis* populations** Genetic diversity of the sampled *G. capensis* populations showing the haplotype diversity, nucleotide diversity and Fu’s F values in each population for the cytochrome *b*/control region datasets. For the Fu’s F values n.s. = non-significant,* = p<0.05, ** = p<0.01, *** = p<0.001. An indication is given where the analysis could not be performed due to “a” too few samples from that population and “b” all individuals within that population having the same haplotype.

|  | **Haplotype diversity** | **Nucleotide diversity** | **Fu's Fs** |
| --- | --- | --- | --- |
| Nieuwoudt-ville | 0.000/0.000 | 0.000/0.000 | a/a |
| Citrusdal | 0.554/0.554 | 0.003/0.062 | 4.271*/13.215*** |
| Moorreesburg | 0.417/0.423 | 0.003/0.030 | 4.297*/11.289*** |
| Darling | 0.600/0.760 | 0.001/0.039 | -2.685*/5.199* |
| Wolseley | 0.787/0.795 | 0.003/0.072 | -0.372^n.s.^/5.077* |
| Ceres | 0.714/0.812 | 0.002/0.061 | 1.887^n.s.^/9.817*** |
| Paarl | 0.471/0.000 | 0.000/0.000 | 1.215^n.s.^/b |
| Worcester | 0.000/0.264 | 0.000/0.028 | b/5.748* |
| Cape Town | 0.640/0.800 | 0.001/0.068 | 2.016^n.s.^/6.890** |
| Struisbaai | 0.442/0.442 | 0.002/0.045 | 5.788*/21.717*** |
| Swellendam | 0.000/0.190 | 0.000/0.001 | b/2.248^n.s.^ |
| Oudshoorn | 0.000/0.000 | 0.000/0.000 | b/b |
| Nottingham Road | 0.667/0.667 | 0.001/0.055 | a/a |
| Wakkerstroom | 0.200/0.200 | 0.002/0.013 | 5.217*/14.208*** |
| Belfast | 0.667/0.667 | 0.001/0.023 | a/a |
