# Supplementary material for: Spatial genetic diversity in the Cape mole-rat, Georychus capensis: Extreme isolation of populations in a subterranean environment
Source: PLoS One. 2018 Mar 15;13(3):e0194165. doi: 10.1371/journal.pone.0194165 (PMC5854370; doi:10.1371/journal.pone.0194165)
Supplement: S3 Table — Pairwise ɸST values between the sampled G. capensis populations with values based on cytochrome b below the diagonal and values based on the control region above the diagonal. n.s. = non-significant,* = p<0.05, ** = p<0.01, *** = p<0.001. (DOCX) [file pone.0194165.s003.docx]

**S3 Table Genetic structure between *G. capensis* populations (cytochrome *b* and control region separately)** Pairwise ɸ_ST_ values between the sampled *G. capensis* populations with values based on cytochrome *b* below the diagonal and values based on the control region above the diagonal. n.s. = non-significant,* = p<0.05, ** = p<0.01, *** = p<0.001.

|  | Nieuwoudt-ville | Citrusdal | Moorreesburg | Darling | Wolseley | Ceres | Paarl | Worcester | Cape Town | Struisbaai | Swellendam | Oudshoorn | Nottingham Road | Wakkerstroom | Belfast |
| --- | --- | --- | --- | --- | --- | --- | --- | --- | --- | --- | --- | --- | --- | --- | --- |
| Nieuwoudt-ville | - | 0.600** | 0.766** | 0.731** | 0.397* | 0.550** | 1.000** | 0.718* | 0.603** | 0.763** | 0.994** | 1.000** | 0.925^n.s.^ | 0.969** | 0.967^n.s.^ |
| Citrusdal | 0.751** | - | 0.731*** | 0.518*** | 0.184** | 0.294*** | 0.658*** | 0.534*** | 0.659*** | 0.724*** | 0.708*** | 0.941*** | 0.797*** | 0.851*** | 0.793*** |
| Moorreesburg | 0.798** | 0.766*** | - | 0.784*** | 0.663*** | 0.731*** | 0.874*** | 0.787*** | 0.380*** | 0.831*** | 0.809*** | 0.971*** | 0.902*** | 0.914*** | 0.903*** |
| Darling | 0.957** | 0.890*** | 0.116*** | - | 0.500*** | 0.498*** | 0.597*** | 0.480*** | 0.711*** | 0.771*** | 0.847*** | 0.962*** | 0.875*** | 0.910*** | 0.873*** |
| Wolseley | 0.631** | 0.364*** | 0.720*** | 0.860*** | - | 0.221*** | 0.557*** | 0.448*** | 0.606*** | 0.672*** | 0.650*** | 0.930*** | 0.777*** | 0.830*** | 0.775*** |
| Ceres | 0.785** | 0.686*** | 0.773*** | 0.904*** | 0.464*** | - | 0.591*** | 0.486*** | 0.670*** | 0.687*** | 0.690*** | 0.942*** | 0.817*** | 0.857*** | 0.812*** |
| Paarl | 0.975** | 0.890*** | 0.120* | 0.183*** | 0.856*** | 0.907*** | - | 0.577*** | 0.758*** | 0.841*** | 0.995*** | 1.000*** | 0.986*** | 0.979*** | 0.994*** |
| Worcester | 1.000** | 0.899*** | 0.347*** | 0.672*** | 0.865*** | 0.917*** | 0.804*** | - | 0.663*** | 0.766*** | 0.890*** | 0.980*** | 0.908** | 0.933*** | 0.914** |
| Cape Town | 0.937** | 0.882*** | 0.334*** | 0.495*** | 0.855*** | 0.895*** | 0.528*** | 0.705*** | - | 0.764*** | 0.685*** | 0.933*** | 0.796*** | 0.840*** | 0.795*** |
| Struisbaai | 0.966** | 0.959*** | 0.951*** | 0.976*** | 0.953*** | 0.965*** | 0.976*** | 0.977*** | 0.972*** | - | 0.878*** | 0.962*** | 0.867*** | 0.896*** | 0.872*** |
| Swellendam | 1.000** | 0.804*** | 0.809*** | 0.962*** | 0.640*** | 0.765*** | 0.980*** | 1.000*** | 0.945*** | 0.981*** | - | 0.999*** | 0.981*** | 0.972*** | 0.989*** |
| Oudshoorn | 1.000** | 0.983*** | 0.975*** | 0.994*** | 0.979*** | 0.987*** | 0.997*** | 1.000*** | 0.991*** | 0.986*** | 1.000*** | - | 0.989*** | 0.989*** | 0.996*** |
| Nottingham Road | 0.992* | 0.979*** | 0.972*** | 0.993*** | 0.974*** | 0.984*** | 0.995*** | 0.998*** | 0.989*** | 0.982*** | 0.999*** | 0.999*** | - | 0.929*** | 0.805^n.s.^ |
| Wakkerstroom | 0.985** | 0.982*** | 0.977*** | 0.989*** | 0.979*** | 0.984*** | 0.990*** | 0.990*** | 0.987*** | 0.982*** | 0.992*** | 0.991*** | 0.978*** | - | 0.890*** |
| Belfast | 0.993* | 0.981*** | 0.974*** | 0.993*** | 0.976*** | 0.985*** | 0.995** | 0.999*** | 0.990*** | 0.982*** | 0.999*** | 0.999*** | 0.985^n.s.^ | 0.786*** | - |
