# Supplementary material for: Spatial genetic diversity in the Cape mole-rat, Georychus capensis: Extreme isolation of populations in a subterranean environment
Source: PLoS One. 2018 Mar 15;13(3):e0194165. doi: 10.1371/journal.pone.0194165 (PMC5854370; doi:10.1371/journal.pone.0194165)
Supplement: S4 Table — Genetic diversity of the B. suillus populations in [4] populations showing the haplotype diversity, nucleotide diversity and Fu’s F values in each population for the cytochrome b/control region datasets. For the Fu’s F values n.s. = non-significant,* = p<0.05, ** = p<0.01, *** = p<0.001. (DOCX) [file pone.0194165.s004.docx]

**S4 Table Genetic diversity of *B. suillus* populations** Genetic diversity of the *B. suillus* populations in [4] populations showing the haplotype diversity, nucleotide diversity and Fu’s F values in each population for the cytochrome *b*/control region datasets. For the Fu’s F values n.s. = non-significant,* = p<0.05, ** = p<0.01, *** = p<0.001.

|  | **Haplotype diversity** | **Nucleotide diversity** | **Fu's Fs** |
| --- | --- | --- | --- |
| Redelinghuys | 0.848/0.836 | 0.006/0.024 | 1.879^n.s.^/6.547** |
| Dwarskersbos | 0.747/0.958 | 0.003/0.029 | -0.885 ^n.s.^/0.149^n.s.^ |
| Sterkfontein | 0.837/0.868 | 0.003/0.026 | 0.025 ^n.s.^/1.458^n.s.^ |
| Piketberg | 0.200/0.380 | 0.001/0.006 | 1.694 ^n.s.^/3.087^n.s.^ |
| Vredenburg | 0.874/0.911 | 0.005/0.037 | -0.048 ^n.s.^/1.092^n.s.^ |
| Cape Town | 0.922/0.928 | 0.003/0.021 | -0.805 ^n.s.^/0.727^n.s.^ |
| Stanford | 0.455/0.773 | 0.002/0.015 | 0.214 ^n.s.^/1.880^n.s.^ |
| Struisbaai | 0.442/0.816 | 0.001/0.006 | -1.326 ^n.s.^/-2.750* |
| Riversdale | 0.721/0.795 | 0.001/0.020 | -0.285 ^n.s.^/3.632* |
| Sedgefield | 0.756/0.644 | 0.002/0.019 | -0.990 ^n.s.^/9.359*** |
