# Supplementary material for: Spatial genetic diversity in the Cape mole-rat, Georychus capensis: Extreme isolation of populations in a subterranean environment
Source: PLoS One. 2018 Mar 15;13(3):e0194165. doi: 10.1371/journal.pone.0194165 (PMC5854370; doi:10.1371/journal.pone.0194165)
Supplement: S5 Table — Pairwise ɸST values between the sampled B. suillus populations in [4] with values based on cytochrome b below the diagonal and values based on the control region above the diagonal. n.s. = non-significant,* = p<0.05, ** = p<0.01, *** = p<0.001. (DOCX) [file pone.0194165.s005.docx]

**S5 Table Genetic structure between *B. suillus* populations (cytochrome *b* and control region separately)** Pairwise ɸ_ST_ values between the sampled *B. suillus* populations in [4] with values based on cytochrome *b* below the diagonal and values based on the control region above the diagonal. n.s. = non-significant,* = p<0.05, ** = p<0.01, *** = p<0.001.

|  | Redelinghuys | Dwarskersbos | Sterkfontein | Piketberg | Vredenburg | Cape Town | Stanford | Struisbaai | Riversdale | Sedgefield |
| --- | --- | --- | --- | --- | --- | --- | --- | --- | --- | --- |
| Redelinghuys | - | 0.383*** | 0.463*** | 0.643*** | 0.386*** | 0.713*** | 0.721*** | 0.940*** | 0.861*** | 0.848*** |
| Dwarskersbos | 0.454*** | - | 0.084*** | 0.288*** | 0.230*** | 0.638*** | 0.634*** | 0.928*** | 0.836*** | 0.819*** |
| Sterkfontein | 0.480*** | 0.112* | - | 0.399*** | 0.269*** | 0.711*** | 0.724*** | 0.946*** | 0.872*** | 0.865*** |
| Piketberg | 0.610*** | 0.297*** | 0.366*** | - | 0.391*** | 0.815*** | 0.857*** | 0.977*** | 0.928*** | 0.934*** |
| Vredenburg | 0.420*** | 0.368*** | 0.412*** | 0.567*** | - | 0.578*** | 0.555*** | 0.907*** | 0.803*** | 0.775*** |
| Cape Town | 0.650*** | 0.736*** | 0.745*** | 0.851*** | 0.674*** | - | 0.439*** | 0.952*** | 0.887*** | 0.879*** |
| Stanford | 0.657*** | 0.770*** | 0.777*** | 0.912*** | 0.685*** | 0.465*** | - | 0.968*** | 0.909*** | 0.907*** |
| Struisbaai | 0.909*** | 0.944*** | 0.942*** | 0.978*** | 0.918*** | 0.952*** | 0.969*** | - | 0.956*** | 0.960*** |
| Riversdale | 0.906*** | 0.941*** | 0.940*** | 0.973*** | 0.913*** | 0.950*** | 0.966*** | 0.9621*** | - | 0.758*** |
| Sedgefield | 0.877*** | 0.926*** | 0.925*** | 0.972*** | 0.891*** | 0.937*** | 0.960*** | 0.958*** | 0.765*** | - |
