# Supplementary figures and images for: Spatial genetic diversity in the Cape mole-rat, Georychus capensis: Extreme isolation of populations in a subterranean environment
Source: PLoS One. 2018 Mar 15;13(3):e0194165. doi: 10.1371/journal.pone.0194165 (PMC5854370; doi:10.1371/journal.pone.0194165)

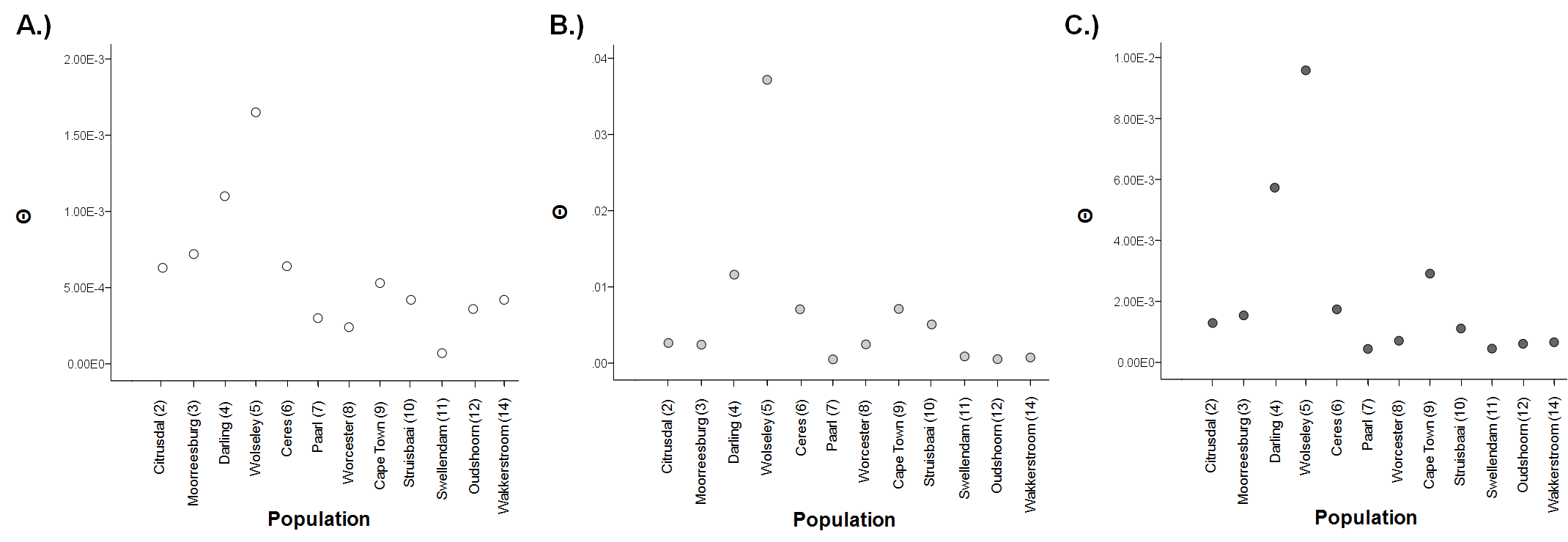

Supplement: S1 Fig — Plot showing the Θ values for the sampled G. capensis populations based on the A) cytochrome b dataset, B) control region dataset and C) combined (cytochrome b control region) dataset. (TIF) [file pone.0194165.s006.tif]
